# Analysis of mRNA-derived siRNAs in mutants of mRNA maturation and surveillance pathways in *Arabidopsis thaliana*

**DOI:** 10.1038/s41598-022-05574-4

**Published:** 2022-01-27

**Authors:** Michal Krzyszton, Joanna Kufel

**Affiliations:** 1 Laboratory of Seeds Molecular Biology, Institute of Biochemistry and Biophysics, Polish Academy of Sciences, Pawinskiego 5a, 02-106 Warsaw, Poland; 2 Faculty of Biology, Institute of Genetics and Biotechnology, University of Warsaw, Pawinskiego 5a, 02-106 Warsaw, Poland

**Keywords:** Plant molecular biology, RNA metabolism, RNAi

## Abstract

Defects in RNA maturation and RNA decay factors may generate substrates for the RNA interference machinery. This phenomenon was observed in plants where mutations in some RNA-related factors lead to the production of RNA-quality control small interfering RNAs and several mutants show enhanced silencing of reporter transgenes. To assess the potential of RNAi activation on endogenous transcripts, we sequenced small RNAs from a set of *Arabidopsis thaliana* mutants with defects in various RNA metabolism pathways. We observed a global production of siRNAs caused by inefficient pre-mRNA cleavage and polyadenylation leading to read-through transcription into downstream antisense genes. In addition, in the *lsm1a lsm1b* double mutant, we identified *NIA1*, *SMXL5*, and several miRNA-targeted mRNAs as producing siRNAs, a group of transcripts suggested being especially sensitive to deficiencies in RNA metabolism. However, in most cases, RNA metabolism perturbations do not lead to the widespread production of siRNA derived from mRNA molecules. This observation is contrary to multiple studies based on reporter transgenes and suggests that only a very high accumulation of defective mRNA species caused by specific mutations or substantial RNA processing defects trigger RNAi pathways.

## Introduction

Small RNA interference (RNAi) pathways are conserved mechanisms implicated in the defence against invading nucleic acids and in the regulation of gene expression. These processes are extensively studied in a model plant species *Arabidopsis thaliana,* where different small RNA pathways can be distinguished based on the nature of small RNA (sRNA) precursors, processing enzymes, and effector complexes. Major plant small RNA classes include microRNA (miRNA), small interfering RNA (siRNA), *trans*-acting siRNA (ta-siRNA), natural antisense transcripts siRNA (nat-siRNA), and heterochromatic siRNA (het-siRNA) (reviewed in^1^). We recently described two mutants involved in mRNA metabolism with elevated production of siRNAs from protein-coding genes^2,3^. Here, we tried to determine if such a phenomenon could also be observed in a broader set of RNA-related mutants.

Arabidopsis genome is relatively small and densely packed with genes, which makes regulation of Pol II transcription especially important. We and others showed that XRN3 5′–3′ exoribonuclease is a crucial factor for Pol II transcription termination and defects in this process give rise to read-through transcripts^2,4^. In the case these transcripts are antisense to mRNAs, they may trigger double-stranded RNA (dsRNA) formation and, consequently, siRNA synthesis^2,5^. Transcripts arising from defective maturation or impaired degradation are considered aberrant. Their features include, but are not limited to, lack of cap structure, as is the case for read-through transcripts in the *xrn3* mutant, lack or shortened poly(A) tail, defective pre-mRNA 3′ end formation and incomplete splicing^6^. It has been postulated that mRNA surveillance and RNAi pathways compete in removing such faulty RNA molecules^6^. Several studies have demonstrated the existence of a new class of sRNAs called rqc-siRNA (RNA quality control small interfering RNA)^7^ or ct-siRNA (coding transcript-derived siRNAs)^8^, which are generated mainly due to defects in RNA degradation machinery from mRNAs that do not normally produce siRNAs. In line with this, our recent work demonstrated a strong accumulation of siRNAs in the mutant of the *DXO1* gene involved in mRNA cap surveillance^3^.

Mutations in factors directly engaged in mRNA decapping^3,7,9–11^ or 5′–3′ and 3′-5′ RNA degradation^8,12–18^ initiate production of siRNAs from mRNAs mediated by RNA-dependent RNA polymerase (RDR) and Dicer-like proteins, mainly but not exclusively by RDR6 and DCL2/4 (reviewed in^6,19^). It has been demonstrated that severe phenotypes of decapping *dcp2* and *vcs* mutants and the RNA degradation double mutant *ski2 xrn4* are partially suppressed by elimination of siRNA production from several hundreds of mRNAs^7,8,18^, which underscores the significance of this new class of siRNAs. These RNA degradation factors and many others have also been identified as suppressors of transgene silencing mediated by siRNA^7,16,20–26^. However, it seems that transgene high expression renders them more sensitive to any disturbances in RNA metabolism than most endogenous mRNAs. Except for mRNA decapping, biogenesis of rqc-siRNA from endogenous mRNAs is strongly increased only when both 5′ and 3′ cytoplasmic RNA degradation pathways are defective, while a disturbance in either of these mechanisms results in an enhanced accumulation of small RNAs from a limited number of loci or reporter transgenes^8,12–15^. This is also true in the L*er* accession of *A. thaliana,* which has an active form of *SOV* (SUPPRESSOR OF VCS) gene, encoding a homolog of DIS3L2 3′–5′ cytoplasmic exonuclease^9,27^. Notably, although a point mutation in the *RRP4* gene of the exosome core complex subunit increases transgene silencing^28^, high-throughput sequencing of small RNAs from the *RRP4 RNAi* line showed a minor impact on the production of siRNAs from mRNAs^29^. Also, aberrant mRNA biogenesis may lead to the production of new siRNAs and the increase of transgene silencing. Enhancement of transgene silencing was observed in the case of defects in mRNA splicing^30,31^, cleavage and polyadenylation^30,32,33^, transcription regulation^16^ and termination^5^. A whole plethora of RNA maturation and degradation mutants showing stronger transgene silencing led to the hypothesis that defects in mRNA metabolism give rise to accumulation of aberrant mRNA species and production of unwanted siRNAs, with a potentially toxic effect on cellular functions^6^.

Here, we set out to validate this assumption by sequencing small RNA libraries from a set of mutants, which potentially are candidates for rqc-siRNA production. Our analysis of *cstf64-2* transcription termination mutant showed a more robust siRNA accumulation from about a thousand mRNAs, mainly due to read-through transcription from downstream convergent genes. However, sequencing of small RNAs from mutants in other factors involved in pre-mRNA 3′ end formation (*rsr1-2*, *fy-2*), mRNA poly(A) tail control (*ahg1-2*, *ccr4a*, *pab2 pab4*), NMD (*upf1-5*, *upf3-1*), RNA 5′–3′ decay (*lsm1a lsm1b*) and splicing (*ncb-4*) revealed that the majority showed only weak or no signs of increased siRNA production from mRNAs. These results suggest that different RNA degradation pathways may substitute for each other or that the rqc-siRNA generating mechanisms are limited only to a specific group of aberrant transcripts.

## Results and discussion

### Strong defects in mRNA cleavage and polyadenylation induce siRNAs production

**Figure 1:**
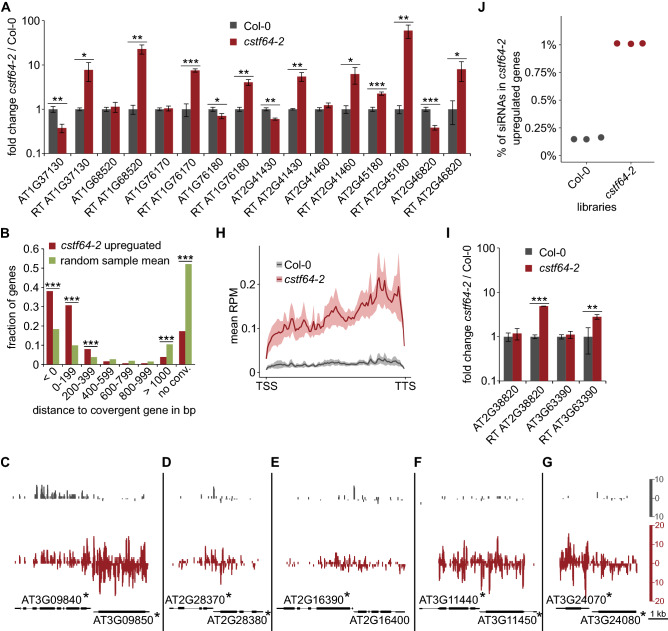
The *cstf64-2* mutation causes defects in Pol II transcription termination and generation of rqc-siRNAs from convergent genes. **(A)** The *cstf64-2* mutant has defects in Pol II termination. The expression level of genes and their downstream regions (marked RT for read-through) as measured by RT-qPCR. Fold changes, expressed relative to the wild-type, represent a mean of three independent biological replicates with standard deviations (s.d.); *P < 0.05; **P < 0.01; ***P < 0.001 (t-test). *ACT2* mRNA was used as a reference. (**B**) The majority of genes producing siRNAs in the *cstf64-2* mutant have convergent gene partners. Genes with siRNA production in the mutant (n = 1102) were divided according to the presence of and distance to the convergent gene. For comparison permutation testing (1 million repetitions) of all protein-coding genes was performed. In each repetition, a set of protein-coding genes was randomly sampled to obtain 1102 genes that were divided as for *cstf64-2*-affected genes. The mean values for each gene group were calculated for all samples, and the frequency of finding samples with the number of genes higher or lower than the true number of affected genes was identified (***P < 10^−6^). **(C–G)** Profiles of small RNA reads in Col-0 and the *cstf64-2* mutant over representative genes with the accumulation of siRNAs in the mutant. Asterisks denote genes with the significant increase of siRNAs identified in small RNA-seq analysis. Small RNAs tracks were normalized to reads per ten million. (**H**) Novel siRNAs produced in the *cstf64-2* mutant are spread along source genes. Mean values of normalised (RPM) siRNA counts was profiled in 10 bp bins over length adjusted protein-coding genes showing siRNA production in the *cstf64-2* mutant (n = 1100; two genes with atypical siRNA peaks in Col-0: AT2G17305 and AT5G41765 were excluded). Shaded areas show 0.95 confidence intervals. (**I**) Genes without converged partners also produce read-through transcripts. The expression level of genes and their downstream regions as measured by RT-qPCR. Fold changes, expressed relative to the wild-type, represent a mean of three independent biological replicates with standard deviations (s.d.); **P < 0.01; ***P < 0.001 (t-test). *ACT2* mRNA was used as a reference. (**J**) Fraction of small RNA reads for *cstf64-2*-affected genes in sequenced libraries.

Our previous work showed that Pol II transcription termination defects in *xrn3-8* mutant lead to small RNA production sourcing from convergent protein-coding gene pairs^2^. The crucial step of Pol II transcription termination is pre-mRNA 3′ end processing conducted by the multisubunit cleavage and polyadenylation complex (CPA). One of its components is the CstF64 protein responsible for the recognition of GU-rich elements in nascent RNAs^34^. We performed small RNA profiling of the *cst64-2* mutant, which exhibits delayed Pol II release due to dysfunction of the cleavage and polyadenylation machinery. Mutant plants are sterile and show characteristic morphology^35^, allowing for the selection of 21 day-old *cstf64-2* homozygous plants grown on soil along with Col-0 wild-type. Since widespread defects in Pol II transcription termination due to the *cstf64-2* mutation have not been directly demonstrated in Arabidopsis, we checked the level of read-through transcripts downstream of several genes that we previously reported to have deficient termination in the *xrn3-8* mutant^2^. All tested regions showed significant up-regulation of transcripts that was often unrelated to changes in the expression of upstream mRNAs (Fig. 1A). This analysis confirmed the role of CSTF64 in Pol II transcription termination and showed that the production of read-through transcripts in the mutant does not always affect the expression of parental genes.

Small RNA libraries were prepared in triplicates and sequenced to 10 million reads per sample (Table S1). Reads were mapped and counted in exons of genes from Araport11 annotation^36^, and counts were used for differential analysis using DESeq2^37^. In such an approach, we focused on the identification of genes with siRNA production as a way to identify the role of their properties in this process. We identified 1172 genes with significantly elevated levels of small RNAs in the mutant (FDR < 0.05, log2FC > 1; Supplementary Dataset 1). Since the CPA complex is mainly involved in the 3′ end formation of pre-mRNAs, we excluded pseudogenes, transposons and other non-protein-coding genomic features, leaving 1102 genes for further analysis. As many as 76% of genes with up-regulated siRNA production in *cstf64-2* plants have antisense convergent genes at a distance of less than 400 bp, which is twice as much as in the random sampling control (Fig. 1B). This observation supports our hypothesis that defects in transcription termination from downstream convergent genes create antisense read-through transcripts. Such RNA species may form double-stranded RNAs by pairing with mRNAs, which triggers siRNA production. This outcome mainly applies to highly expressed gene pairs since low-expression genes would not generate abundant read-through transcripts that could initiate siRNA synthesis. Examples of such gene pairs are shown in Fig. 1C–G. Consistently, genes with increased siRNAs show high overlap with those identified in the *xrn3-8* transcription termination-deficient mutant^2^ and low with mutants involved in cytoplasmic mRNA degradation^7,8^ (Supplementary Fig. 1A–C). Contrary to our expectations, we do not observe an enrichment of small RNAs exclusively at 3′ ends of affected genes (Fig. 1H). This suggests that initial dsRNA formation close to the 3′ end of the transcript may trigger siRNA production from the whole molecule, which requires RNA-dependent RNA (RDR) polymerase activity. Another possibility is that defective mRNA 3′ end formation is a feature that recruits RDR independently of antisense transcripts pairing. Such a mechanism has been demonstrated earlier for several transgene reporters^32,33^. In our data, it is supported by identification of genes that lack downstream convergent partners but still generate read-through transcripts and produce siRNAs (Fig. 1B,I).

**Figure 2:**
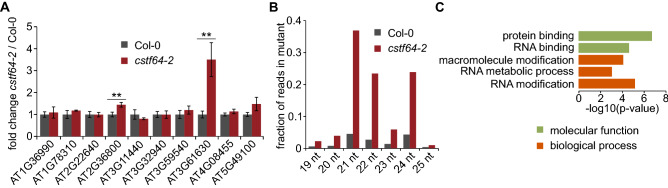
Gene expression changes in the *cstf64-2* mutant. (**A**) RT-qPCR analysis of selected genes shows that accumulation of rqc-siRNAs in *cstf64-2* mutant does not result in down-regulation of their source mRNAs. Fold changes, expressed relative to the wild-type, represent a mean of three independent biological replicates with standard deviations (s.d*.*); **P < 0.01 (t-test). *ACT2* mRNA was used as a reference. **(B)** Length distribution of siRNAs from protein-coding genes showing their up-regulation in the *cstf64-2* mutant. (**C**) GO terms enriched among protein-coding genes with enhanced siRNA production in the *cstf64-2* mutant. Shown are GO terms with FDR < 0.001 and enrichment > 1.5. Colour legend shows GO terms ontologies.

Albeit widespread among genes, the accumulation of novel siRNAs appears to be relatively moderate in terms of the absolute number of sRNAs (Fig. 1H). Small RNAs from genes showing an increase in their level in *cstf64-2* represent 1% of mutant small RNA libraries and 0.15% of wild-type libraries (Fig. 1J). Such a limited increase may explain why siRNA accumulation does not lead to significant changes in the expression of their source genes, as assayed for ten mRNAs by RT-qPCR (Fig. 2A). However, we cannot exclude that some genes that have not been tested respond to these siRNAs. The length distribution of small RNAs from genes with siRNA increase in *cstf64-2* plants has two prominent peaks—21 and 24 nt (Fig. 2B). Since it is assumed that biogenesis of 24 nt long siRNAs takes place in the nucleus and this class is involved in transcriptional gene silencing^1^, novel siRNAs in *cstf64-2* plants could be produced mainly in this cellular compartment. It could also explain the lack or only weak impact of sRNA increase on the level of cytoplasmic mRNAs. Finally, GO term enrichment analysis for genes producing more siRNAs in the *cstf64-2* mutant showed that a significant number are implicated in mRNA metabolism (Fig. 2C). This observation suggests a self-regulatory mechanism that affects gene expression when read-through transcripts accumulate in physiological conditions, as recently described for drought stress in Arabidopsis^38^. In plants, siRNAs derived from natural antisense transcripts have been described to be involved in the regulation of expression of their source genes^39,40^. It is possible that the controlled production of read-through transcripts followed by siRNA accumulation may affect the pattern of gene expression on the local scale or genome-wide.

### The majority of tested mutants show limited production of mRNA-derived siRNAs

We chose the *CSTF64* gene for our small RNA analysis as its mutation shows a clear developmental phenotype^35^, suggesting the importance of this gene for plant RNA metabolism. Two other components of the 3′ end processing machinery have been reported to act as suppressors of transgene silencing by siRNAs, *CSTF64* paralog *ESP1/RSR1* and CPSF100 component of the CPA complex^30^. Therefore, we decided to extend our analysis to include the viable *rsr1-2* knock-out mutant^41^ and the hypomorphic mutant of the *FY* gene encoding a protein that interacts with CPSF100 in the CPA complex^30^. Since mRNA 3′ end formation and polyadenylation efficiency may affect its fate, we decided to add mutants of factors potentially involved in poly(A) tail length control. Importantly, two Arabidopsis putative deadenylating enzymes PARN/AHG2 and CCR4a act as suppressors of transgene silencing dependent on RDR6 and SGS3 (SUPPRESSOR OF GENE SILENCING3) and co-localise with siRNA-bodies^24^. However, it should be emphasised that data on the cellular localisation of AHG2 is not consistent with its function in mitochondria^42^. In turn, Poly(A)-binding (PAB) proteins regulate mRNA deadenylation and translation, contributing to mRNA cytoplasmic turnover and stability^43^. Out of eight Arabidopsis PAB proteins, PAB2, PAB4 and PAB8 are expressed in vegetative tissues. While triple mutants are not viable, the combination of *pab2* and *pab4* knock-out mutations shows the strongest phenotypic defects^44^.

**Figure 3:**
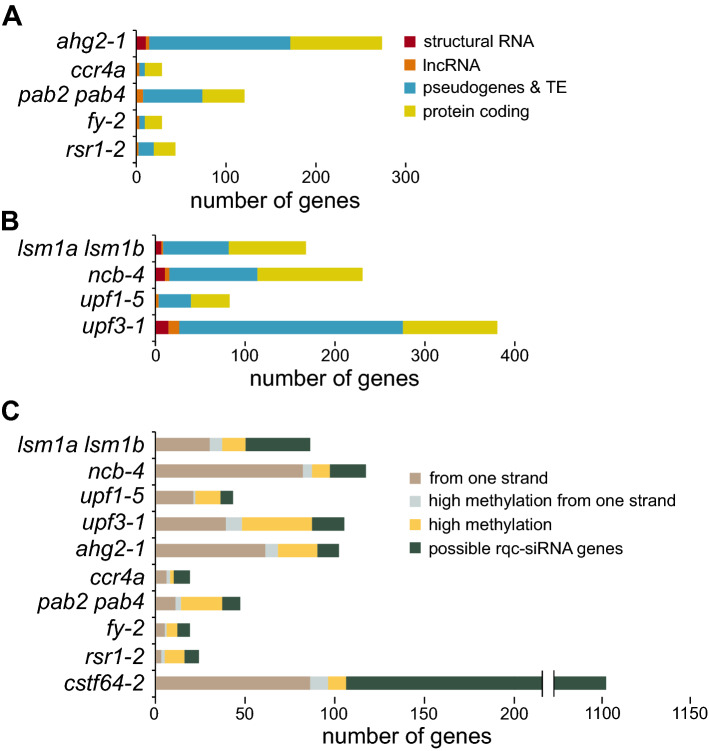
The majority of analysed mutants show limited production of rqc-siRNAs. **(A,B)** Araport11-annotated genes with significant up-regulation of small RNAs (log_2_FC > 1, FDR < 0.05) were divided into those encoding structural RNAs (tRNA and snRNA), long non-coding RNAs, pseudogenes and transposable elements, and protein-coding. **(C)** Protein-coding genes with the accumulation of siRNAs were divided into those that produce siRNAs only from one strand, genes with high DNA methylation level (> 20% of all cytosines), genes fitting these two categories and genes that represent a possible source of rqc-siRNAs.

Small RNA libraries were prepared in biological triplicates from 14 day-old Col-0 and *ahg2-1*, *ccr4a*, *rsr1-2*, *fy-2* and *pab2 pab4* mutant seedlings grown on MS solid medium, sequenced (Table S1) and analysed as described above. We identified only from 28 (*crr4a* and *fy-2*) to 273 (*ahg2-1*) genes with significantly elevated levels of small RNAs (FDR < 0.05, log2FC > 1; Fig. 3A, Supplementary Dataset 1). However, these numbers dropped to 19 and 102, respectively, when only protein-coding genes were considered (Fig. 3A). This was caused by a large fraction of affected genes belonging to pseudogenes and transposable elements. Since the analysed mutations are predicted to affect mRNAs, these effects are most likely indirect. Due to a small number of genes with changes in sRNA level, principal component analysis (PCA) showed poor separation of genotypes on the plot (Supplementary Fig. S2A).

For *rsr1-2* and *fy-2* mutants predicted to be involved in pre-mRNA cleavage and polyadenylation, we checked the level of read-through transcripts downstream of five genes with prominent defects in *cstf64-2* and *xrn3-8* mutants (Supplementary Fig. S2B). All showed a much weaker or no increase in read-through transcription. Lack of termination defect may explain the very limited accumulation of small RNAs in these mutants. Still, five genes with siRNA increase in the *rsr1-2* mutant have antisense partners and were also found in the *cstf64-2* analysis (Supplementary Fig. S2C–G), showing that effects in the *rsr1-2* mutant are similar but more subtle or limited to a specific set of genes.

A very limited number of endogenous mRNAs producing siRNAs in mutants that have been shown to act as suppressors of transgene silencing was unexpected. Therefore, we set out to extend our analysis again by including mutants in factors affecting other aspects of mRNA metabolism. UPF1 and UPF3 proteins are key factors of Nonsense-mediated decay (NMD), which is an important translation-dependent mRNA surveillance mechanism that eliminates aberrant transcripts containing premature translation stop codons and also contributes to the regulation of gene expression (reviewed in^45,46^). Interestingly, *upf1* and *upf3* mutants have been shown to enhance transgene silencing through the production of transgene-derived siRNAs. Moreover, UPF1 protein co-localises with cytoplasmic siRNA-bodies, which represent sites of siRNA production^24^. COILIN is a conserved structural protein required for the assembly of peri-nucleolar organelles called Cajal bodies, which in different organisms are implicated in the formation and function of small nuclear (snRNA), small nucleolar (snoRNA) and small Cajal body-specific RNAs (scaRNA). Consequently, Cajal bodies have a role in mRNA splicing and rRNA maturation (reviewed in^47^). Although in plants composition and role of Cajal bodies are much less studied, they contain COILIN and several components of snRNP complexes including SmD3, SmB, U2A' and U2B''^48–50^. Interestingly, splicing defects have been shown to enhance siRNA production for transgene reporter transcripts^30,51^ and SmD1, which is involved in splicing and has been reported to play a role in transgene silencing^31^. Finally, Arabidopsis LSM1 protein is a subunit of the cytoplasmic heptameric LSM1-7 complex engaged in mRNA decapping and degradation^52,53^. In yeast and Metazoa, mRNA deadenylation and oligouridylation attract the LSM1-7 complex, which recruits decapping proteins that trigger XRN1-mediated RNA degradation from the 5′ end and at the same time protects RNA 3′ end against degradation by the exosome (reviewed in^54^). Although information concerning the role of LSM proteins in mechanisms engaging small RNAs is lacking, XRN4, a plant homologue of yeast Xrn1, acts as a suppressor of silencing by small RNAs, and its absence leads to the accumulation of decapped mRNAs that are targeted for siRNA production^8,20,21,25^.

Small RNA libraries from wild-type and *lsm1a lsm1b*, *upf1-5*, *upf3-1* and *ncb-4* (*coilin*) mutant 14 day-old MS-grown seedlings were prepared, sequenced (Table S1) and analysed as for the previous set of mutants. PCA plot showed that analysed libraries formed separate coherent groups (Supplementary Fig. S3A), however, the differences between genotypes may be modest as the variance in both PC1 and PC2 is small. Consistently, as with the previous set of mutants, we found a limited number of protein-coding genes with increased siRNA production (Fig. 3B). Several works described the accumulation of rqc-siRNAs in mutants with perturbed mRNA degradation. It was proposed that genes producing this class of siRNAs were direct substrates of the mutated factors. We were interested whether any of the protein-coding genes showing an increase in sRNA level in sequenced mutants had the properties of rqc-siRNA source, as it would identify them as the target of the analysed factors.

It has been proposed that small RNAs produced from mRNAs with a high expression and turnover represent degradation by-products as they are generated only from the sense strand and show neither the length distribution nor the first nucleotide bias observed for canonical sRNAs^55^. Synthesis of the sense-strand sRNAs does not involve RNA-dependent polymerases, which are required for rqc-siRNA production^7,8^, and such small RNAs cannot post-transcriptionally down-regulate the expression of their source mRNAs. Therefore, we tested the strandedness of small RNAs from protein-coding genes based on the criterion that the ratio of small RNAs mapping to both strands should be in the range of 0.25–4 for double-strand-derived siRNAs (as described in^7^). Most of the tested mutants showed a significant fraction of genes with small RNAs produced only from one strand (21–74%), except for the *cstf64-2* mutant (Fig. 3C). We analysed changes in mRNA levels for ten of these genes using RT-qPCR, and most of them were up-regulated (Supplementary Fig. S3B). It is consistent with the notion that the accumulation of small RNAs produced exclusively from mRNA strand reflects changes in gene expression. Alternatively, in the case of genes with unaltered expression, mRNAs may be more efficiently converted into small RNAs due to their enhanced degradation. Both scenarios are probably true for several potential NMD substrates, including *SMG7* as well as other mRNAs with an intron in the 3′ UTR (for example, *AT3G03710*, *AT3G27906* and *AT5G54390*) or containing an upstream ORF (for example, *AT1G36730, AT3G18000*)^46^ as they were identified among the genes producing more sRNAs in *upf3* or *upf1* mutants but only from their coding strand (Supplementary Dataset 1).

Another criterion for a genuine rqc-siRNA source is that siRNA accumulation should be attributed to defects in mRNA metabolism and should not spread from pre-existing genomic sRNA production hotspots (Supplementary Fig. S3C–F). Such hotspot genes usually have high levels of siRNA production also in the wild-type and consequently high levels of DNA methylation, which is maintained by sRNA-dependent pathways^1^. We observed that genes with accumulation of sRNAs in mutants, with the exception of *cstf64-2*, tend to produce more small RNAs also in their wild-type controls compared to the average value of all protein-coding genes (Supplementary Fig. 4A–C). Consequently, these small RNA hotspots may have elevated DNA methylation. We used Col-0 DNA methylation data^56^ to check the methylated cytosine fraction in the affected genes and noticed that it is often much higher than the average for all protein-coding genes. This effect was not observed for *dcp2*, *vcs*^7^ and *ski2 xrn4* mutants^8^, which represent the golden standard of mutants producing rqc-siRNAs (Supplementary Fig. 4D). We cannot exclude that genes with high DNA methylation are the true source of rqc-siRNAs, but they would constitute atypical cases as they can be silenced even in wild-type plants. The presence of methylation and siRNA production hotspots in different parts of the gene can also be explained by spreading of small RNA synthesis^57^. Therefore, we decided to filter out genes with high level of DNA methylation using an arbitrarily chosen criterion of methylation level higher than 20% of all cytosines in a given gene, which is six times more than the average for protein-coding genes. Using both the strandedness and hotspot criteria, most libraries showed 53–88% dropouts, except for the *cstf64-2* mutant with only 10% of the excluded genes (Fig. 3C).

Such filtering left a minimal number of mRNAs potentially generating rqc-siRNAs. It has been suggested that rqc-siRNAs are exclusively 21–22 nt long^7,8^. However, protein-coding genes with siRNA accumulation show a prominent peak for siRNAs of 24 nt in most of the tested mutants (Supplementary Fig. 5A). Assuming that restricting the analysis to this class of siRNAs will enhance our results, we repeated the differential analysis for 21–22 nt long small RNAs (Supplementary Dataset 2). As a control, we used 24 nt siRNAs that are implicated in transcriptional gene silencing (Supplementary Dataset 3). The results obtained after eliminating non-protein-coding genes, genes producing small RNAs from only one strand, and from genomic siRNA hotspots (Supplementary Fig. S5C–E) show high overlap with those for small RNAs before size selection (Supplementary Fig. S5F). Surprisingly, restricting the analysis to 21–22 nt small RNAs had only a minor effect on the identification of rqc-siRNA-producing genes.

It appears that there may be only a few examples of genuine rqc-siRNAs produced in the analysed mutants. However, each of the possible candidates requires careful examination to exclude the existence of adjacent siRNA production hotspots or having atypical small RNA profiles (Supplementary Fig. S3C–F). For example, the *fy-2* mutation is characterised by elevated levels of siRNAs derived from the *FY* gene itself, but generated only from the gene fragment downstream of the T-DNA insertion, pointing to the possibility that the aberrant transcript originates from the insert (Supplementary Fig. S6A). Moreover, the *fy-2* mutation causes accumulation of rqc-siRNAs from the *DXO1* gene (Supplementary Fig. S6B) encoding the plant homolog of human DXO, which is involved in mRNA cap surveillance and mRNA degradation^3,10,11^. Lack of Arabidopsis *DXO1* results in a strong up-regulation of rqc-siRNAs from several hundred genes^3,11^. However, the expression of *DXO1* is not altered in *fy-2* plants (Supplementary Fig. S6C), which accordingly do not show strong phenotypes observed for the *dxo1-2* mutant. Therefore, the functional significance of this observation is currently unclear.

It is apparent that in most of the tested mutants only a small fraction of genes become the source of rqc-siRNAs. This can be considered surprising as selected mutants show an increase in transgene silencing or are involved in pathways whose dysfunctions have been suggested to induce rqc-siRNAs production. The enhanced generation of transgene-derived siRNAs is likely due to the particularly high expression of transgenes, making them susceptible to the production of a wide variety of defective transcripts. Their degradation relies on quality control and decay pathways, but they can easily avoid degradation and trigger siRNA synthesis even in wild-type plants. In turn, endogenous aberrant or superfluous transcripts, including those highly expressed, most likely evolved protective mechanisms to avoid such scenarios. They are quickly recognised by specialised quality control pathways and efficiently eliminated without activating RNA interference.

The limited production of rqc-siRNAs in analysed mutants can be explained by case-specific circumstances. The most obvious situation is functional redundancy due to the presence of closely related paralogs that replace the activities missing in individual mutants: PAB8 in the *pab2 pab4* mutant, CCR4b-g in the *ccr4a* mutant and CSTF64 in the *rsr1-2* mutant^30,44,58^. In the latter case, however, RSR1/ESP1 may have a specific role in the CPA complex since it lacks the RRM domain present in CSTF64^30^. Redundancy can also be provided by closely related proteins or complexes with similar functions, as is the case of CCR4-NOT, PAN2-PAN3 and PARN/AHG2 deadenylases. Lack of rqc-siRNA production in *ahg2* mutant may also be attributed to the mitochondrial^42^ rather than cytoplasmic functions of AHG2^24^. In addition, *fy-2* and *upf1-5* mutations are hypomorphic and were used since viable knockout lines are not available. Their remaining functionality may be sufficient to suppress rqc-siRNA production. Finally, specific features of transcripts generated as a result of defects in mRNA metabolism may not trigger rqc-siRNA production. This may be true for NMD substrates that accumulate in *upf1-5* and *upf3-1* mutants, and mRNAs with defective maturation in *ncb-4* and *fy-2* mutants involved in splicing and 3′ end processing, respectively. Nevertheless, we found that some genes may be the source of rqc-siRNA production in *lsm1a lsm1b* double mutant.

### Lack of LSM1 cause accumulation of rqc-siRNAs from known mRNA sources

**Figure 4:**
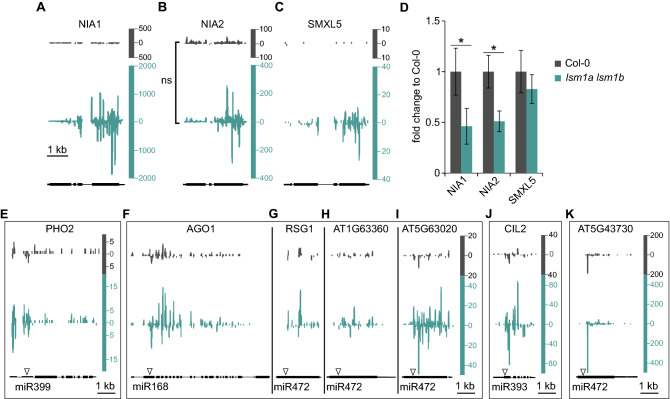
Mutations of *LSM1* genes induce rqc-siRNA production from known hotspots. **(A–C)** Profile of small RNA reads in Col-0 and the *lsm1a lsm1b* mutant over *NIA1*, *NIA2* and *SMXL5* genes. Small RNAs tracks were normalised to reads per ten million. **(D)** RT-qPCR analysis of genes showed in profiles A-C. Fold changes, expressed relative to the wild-type, represent a mean of three independent biological replicates with standard deviations (s.d.); *P < 0.05 (t-test). *UBC9* mRNA was used as a reference. **(E–K)** Profiles of small RNA reads in Col-0 and the *lsm1a lsm1b* mutant over miRNA targets (triangles indicate positions of miRNA cleavage). Small RNAs tracks were normalised to reads per ten million.

We observed a very high accumulation of siRNAs generated from the *NIA1 locus* in the *lsm1a lsm1b* mutant (Fig. 4A). Similar siRNAs were detected in the *ski2 xrn4* double mutant and reported to be responsible for down-regulation of *NIA1* and *NIA2* (*NIA1* paralog) mRNAs and some of the observed mutant phenotypes^8,18^. In contrast, in the *lsm1a lsm1b* mutant, the *NIA2* gene produced only moderately more siRNAs (Fig. 4B), and the change in their level was not statistically significant when small RNAs of all lengths were taken into account, but was prominent for 21–22 nt small RNAs (Supplementary Dataset 2). As expected from the increase of small RNAs in *lsm1a lsm1b* plants, we observed down-regulation of *NIA1* and *NIA2* mRNAs (Fig. 4D), but these plants did not show such severe morphological phenotypes as the *xrn4 ski2* double mutant^8,53^. Another gene reported to accumulate siRNAs with an important outcome for plant physiology is *SMXL5*. High production of siRNAs from *SMXL4* and *SMXL5* induces their silencing and causes over-accumulation of carbohydrates and defects in phloem transport^18,59^. We observed a moderate accumulation of small RNAs from *SMXL5*, but not from *SMXL4*, in the *lsm1a lsm1b* mutant, consequently with no impact on *SMXL5* expression (Fig. 4C,D). The different outcomes of rqc-siRNA production on the level of their source mRNA can be explained by the extent of their accumulation. A high amount of siRNA would lead to reduced gene expression (*NIA1*), while a moderate increase in siRNA would not affect mRNA levels (*SMXL5*). Interestingly, when used as a transgenic reporter, *NIA2* induces silencing of its endo- and exogenous copies^60^, whereas *SMXL5* is one of the six protein-coding genes which produce more siRNAs when *DCL4* is mutated^59^. These observations suggest that a low number of genes accumulating rqc-siRNAs in the *lsm1a lsm1b* mutant may represent those especially sensitive to induction of rqc-siRNA biogenesis. Recent work has shown the potential physiological role of 22 nt siRNAs produced from *NIA1/2* mRNAs as they are induced not only by *xrn4 ski2* mutations but also by nitrogen starvation and ABA treatment^18^.

In the *lsm1a lsm1b* mutant, we also observed a modest siRNAs increase for *AGO1* and several other miRNA targets that produce siRNAs also in wild-type plants (Fig. 4E–K), but the level of these mRNAs was unaffected by the enhanced siRNA production (Supplementary Fig. S7A). On the other hand, miR168 and miR472, but not miR393 and miR399, which are predicted to target these mRNAs, accumulated in the *lsm1a lsm1b* mutant (Supplementary Fig. S7B). As LSM1 potentially cooperates with XRN4 in plant 5′–3′ mRNA decay, siRNAs increase from these genes may reflect the role of XRN4 in removing 3′ fragments of mRNAs targeted by miRNAs^21^ and is consistent with siRNA accumulation from miRNA targets in the *ski2 xrn4* double mutant^8^. However, it is not clear why lack of LSM1 affects a very limited number of miRNA targets and why the levels of only some miRNAs change in the mutant.

Despite the low number of genes generating siRNAs in the absence of the decapping activator LSM1, some of them seem to match the requirements of the rqc-siRNA source, including the length of 21–22 nt of produced sRNAs (Supplementary Fig. S7C). A limited accumulation of rqc-siRNAs resembles single *xrn4*, *cer7* and *ski2* mutants^12–14^, suggesting that only mutations with a strong effect on RNA decay, i.e. decapping or simultaneous inhibition of 5′–3′ and 3′–5′ degradation, are capable of triggering rqc-siRNA production. Moreover, these rqc-siRNAs are mostly limited to known rqc-siRNA hotspots.

## Conclusions

Shortly after the discovery of plant small interfering RNAs^61^, their accumulation under perturbed RNA degradation conditions was reported^20^. Moreover, it has been observed that many defects in mRNA maturation or degradation can lead to the accumulation of aberrant endogenous or transgenic transcripts that are recognised by RNAi pathways^23–25,30,31,62^. More recently, a new class called RNA quality control siRNA^7^ or coding transcript-derived siRNA^8^ was described. However, other experiments have shown that in most cases the number of endogenous protein-coding genes that accumulate rqc-siRNAs is surprisingly low^12–14^. Our work supports these studies by providing evidence based on additional nine mutants with defects in different RNA metabolism pathways. With the exception of the *cstf64* mutation, the remaining mutants showed no or only limited rqc-siRNA synthesis. This strongly suggests that results from transgene-based studies are incompatible with quality control processes that affect endogenous mRNAs. From the set of mutants analysed in this study, only strong impairment of pre-mRNA 3′ end processing and Pol II transcription termination in the *cstf64-2* mutant led to genome-wide production of rqc-siRNAs. Notably, these rqc-siRNAs may also be produced in some physiological conditions as the accumulation of read-through transcripts was reported to be strongly enhanced under drought stress^38^. It was previously proposed that plants evolved multiple RNA quality control pathways, protecting them from the production of unwanted siRNAs. Such mechanisms would allow the distinction between endogenous and exogenous RNA species. The limited production of rqc-siRNAs in different RNA-related mutants supports this hypothesis.

## Methods

Wild-type Col-0 and mutant lines: *ahg2-1*^63^, *ccr4a* (*SAIL_784_A07*)^24^, *pab2 pab4* (*SALK_026293 SALK_113383*)^44^ (these three lines were a kind gift of Hervé Vaucheret, INRA Centre de Versailles-Grignon, France), *cstf64-2* (*SAIL_794_G11*, kind gift of Caroline Dean, John Innes Centre, UK)^35^, *fy-2*^64^ (kind gift of Szymon Swiezewski, IBB, Poland), *lsm1a lsm1b* (*SALK_106536 SAIL_756_305*, obtained in our earlier work)^53^, *ncb-4*^49^ (kind gift of Peter Shaw, John Innes Centre, UK), *rsr1-2* (*SALK_078793*, kind gift of Dietmar Funck, University of Konstanz, Germany)^41^, *upf1-5* and *upf3-1* (*SALK_112922*, *SALK_025175*, kind gift of Brendan Davies, University of Leeds, UK)^65^ were used in this study. We had obtained permission to grow genetically modified plants and we handled them in accordance with the institutional, and national guidelines and legislation. Seeds were sown on MS plates and stratified for 2 days at 4 °C. Plants were grown at constant 21 °C under long-day conditions and harvested at the age of 14 days. The *cstf64-2* mutant was grown in soil at constant 21 °C under long-day conditions, and homozygous 21-day-old plants were harvested based on their phenotype. Wild-type Col-0 plants for this experimental set were grown together at the same time and condition but their small RNA sequencing analysis results are part of the earlier GEO submission (GSE99600). RNA was isolated using the Tri Reagent method and analysed with Bioanalyzer. All library preparation steps using NEB Next Small RNA Library Prep Set for Illumina, including PAGE selection of small RNAs and sequencing with Illumina HiSeq4000 in 50 bp single-end mode, were conducted by BGI Sequencing Services. Obtained fastq files were quality checked using FastQC (v0.10.1; http://www.bioinformatics.babraham.ac.uk/projects/fastqc/) with all the replicates showing high-quality RNA-seq data. Adaptor sequences (Illumina Small RNA Adapter2 TCGTATGCCGTCTTCTGCTTGT) were removed using cutadapt (v1.9.dev6; http://cutadapt.readthedocs.io/en/stable/guide.html), and reads were quality trimmed with sickle se (v0.940; https://github.com/najoshi/sickle) with the following command-line parameters: *-t illumina -q 20 -l 15*. Reads were mapped to the TAIR10 *A. thaliana* genome from Ensembl^66^ (release v29) using bowtie^67^ (v1.0.0) with the following command-line parameters: *-phred33 -v 0 -k 10 -m 10*. Mapped reads were sorted using samtools sort (v1.1)^68^, counted with HTseq-count^69^ (v0.6.0) with or without respect to the strand and using exon features from Araport11 gene annotation release 201604^36^. In that annotation, exon stands for any gene fragment (both protein-coding and non-protein-coding) included in a mature RNA molecule. Differential expression and PCA was performed using DESeq2^37^ (v1.8.2) R (v3.2.2) package with parameter *alpha* = *0.05*. Genes with FDR < 0.05 and absolute log_2_FC > 1 were considered significantly changed. Analysis of siRNAs of different lengths was performed on reads grouped according to their length using reformat.sh with parameters *minlength* and *maxlength* from bbmap v35.x (https://sourceforge.net/projects/bbmap/). For DNA methylation analysis, we extracted the number of methylated cytosines for each gene using an R script and published data for Col-0 ecotype^56^; GSM1085222). Alignment coverage graphs were calculated with genomeCoverageBed from bedtools^70^ (v2.17.0) for all the alignment files with normalisation to the number of reads and were converted to bigwig format with bedGraphToBigWig (v4) from the UCSC Genome Browser application binaries collection (http://hgdownload.cse.ucsc.edu/admin/exe/linux.x86_64/) for visualisation in Integrated Genome Browser^71^. GO enrichment analysis was performed using g:Profiler (https://biit.cs.ut.ee/gprofiler/gost) with FDR < 0.001 and enrichment > 1.5. RT-qPCR was carried out on 2 µg of total RNA following DNase I digestion (TURBO DNase, Thermo Fisher Scientific) with Random Primers and SuperScript III Reverse Transcriptase (Thermo Fisher Scientific). Quantitative PCR was performed using SYBR Green I Master Mix (Roche) using the Roche qPCR platform (LightCycler 480). Results were normalised to *UBC9* or *ACT2* mRNA. Primers sequences used for qPCR are listed in Supplementary Table S2.

## Supplementary Information

Supplementary Information 1.

Supplementary Information 2.

Supplementary Information 3.

Supplementary Information 4.

## Data Availability

The data presented in this study are openly available in the GEO database, reference numbers GSE169171 and GSE99600 (Col-0 wild-type for *cstf64-2* mutant).
