# Supplementary material for: Analysis of mRNA-derived siRNAs in mutants of mRNA maturation and surveillance pathways in Arabidopsis thaliana
Source: Sci Rep. 2022 Jan 27;12:1474. doi: 10.1038/s41598-022-05574-4 (PMC8795450; doi:10.1038/s41598-022-05574-4)
Supplement: Supplementary file 4 — Supplementary Information 4. [file 41598_2022_5574_MOESM4_ESM.pdf]

*Supplementary Figures and Tables*

# Analysis of mRNA-derived siRNAs in mutants of mRNA maturation and surveillance pathways in *Arabidopsis thaliana*

Michal Krzyszton and Joanna Kufel

**Figure S1.** Overlap of genes with enhanced siRNA production in the *cstf64-2* mutant with published data sets.

**Figure S2.** *rsr1-2* and *fy-2* mutants show only limited defects in transcription termination.

**Figure S3.** Examples of non-rqc-siRNA genes.

**Figure S4.** Several possible rqc-siRNA sources are hotspots of siRNA production also in the wild-type.

**Figure S5.** Selection of 21-22 nt siRNAs does not enhance identification of rqc-siRNA genes.

**Figure S6.** Novel sources of siRNAs in the *fy-2* mutant.

**Figure S7.** Lack of LSM1 affects siRNA production from some miRNA target genes.

**Table S1.** Depth of library sequencing.

**Table S2.** List of primers used in this study.

**Supplementary Dataset S1.** List of affected genes based on small RNA-seq (separate file).

**Supplementary Dataset S2.** List of affected genes based on small RNA-seq – analysis for 21-22 nt long siRNAs (separate file).

**Supplementary Dataset S3.** List of affected genes based on small RNA-seq – analysis for 24 nt long siRNAs (separate file).

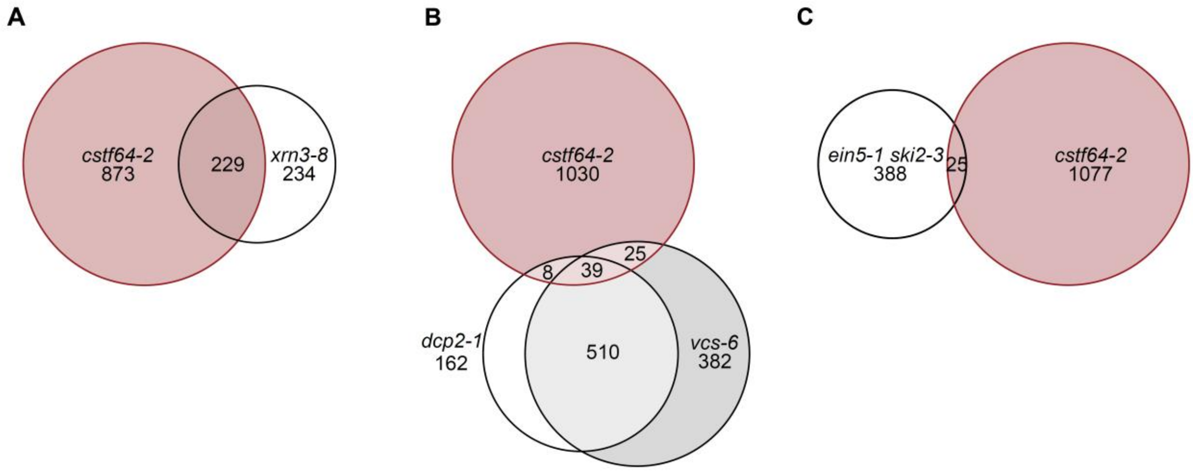


**Figure S1**. Overlap of protein-coding genes with enhanced siRNA production in the *cstf64-2* mutant with published data sets. **(A)** Venn diagram showing overlap with protein-coding genes producing more siRNAs in the transcription termination-deficient *xrn3-8* mutant (Krzyszton et al. 2018). **(B)** Venn diagram showing overlap with protein-coding genes producing more siRNAs in mRNA decapping mutants *dcp2-1* and *vcs-6* (Martinez de Alba et al. 2015). **(C)** Venn diagram showing overlap with protein-coding genes producing more siRNAs in the mRNA cytoplasmic degradation *ein5-1 ski2-3* double mutant (Zhang et al. 2015).

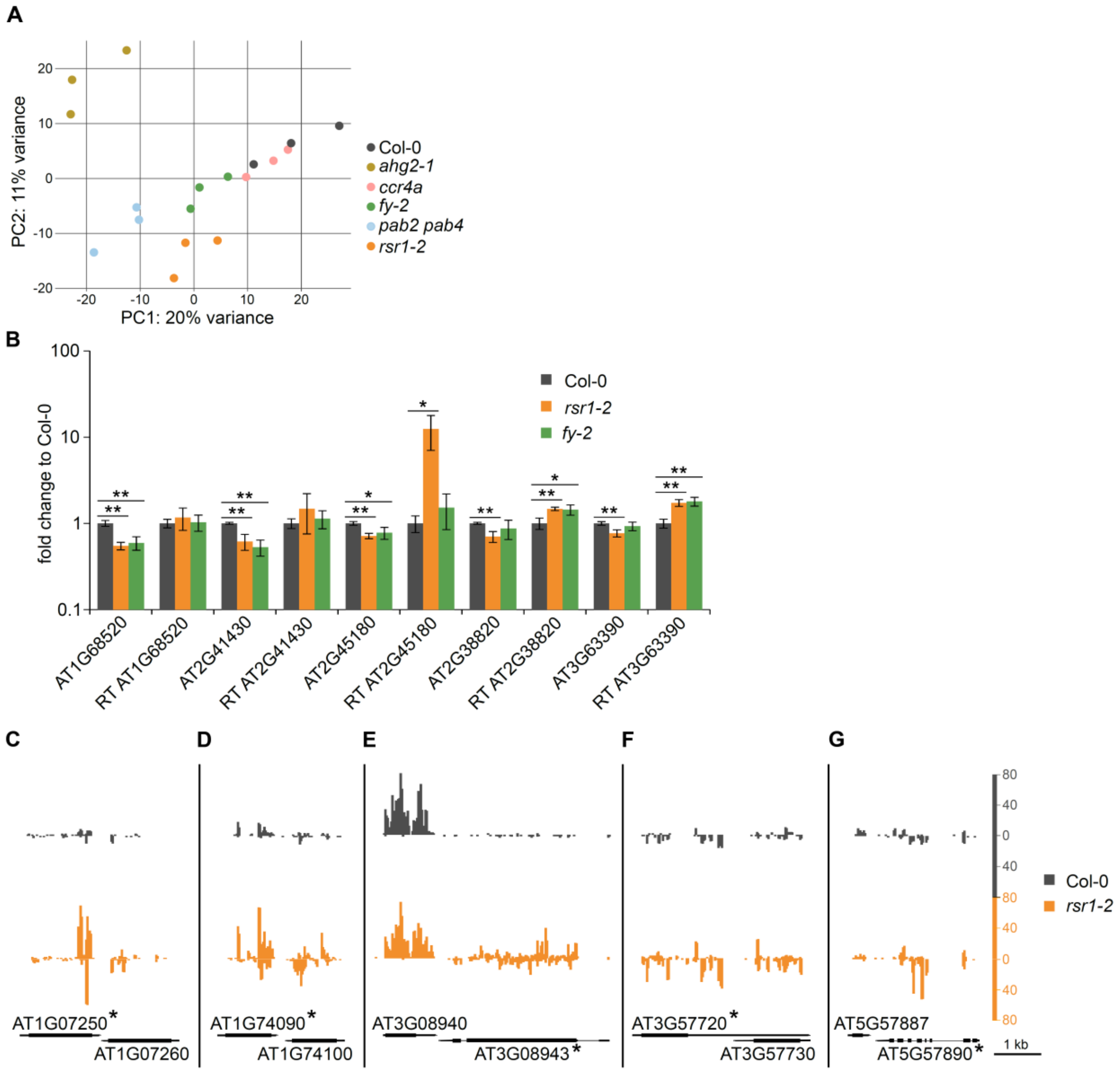


**Figure S2**. **(A)** PCA plot for small RNA-seq for the first set of mutants. **(B)** RT-qPCR for selected genes show that *rsr1-2* and *fy-2* mutations lead to limited defects in Pol II transcription termination. Fold changes, expressed relative to the wild-type, represent a mean of three independent biological replicates with standard deviations (s.d.); **P* < 0.05; ***P* < 0.01 (t-test). *ACT2* mRNA was used as a reference. **(C-G)** Profiles of small RNA reads in Col-0 and the *rsr1-2* mutant over genes with convergent partner and accumulation of siRNAs in the mutant. An asterisk denotes gene from each pair with a significant increase in siRNAs identified in small RNA-seq analysis. Small RNAs tracks were normalised to reads per ten million.

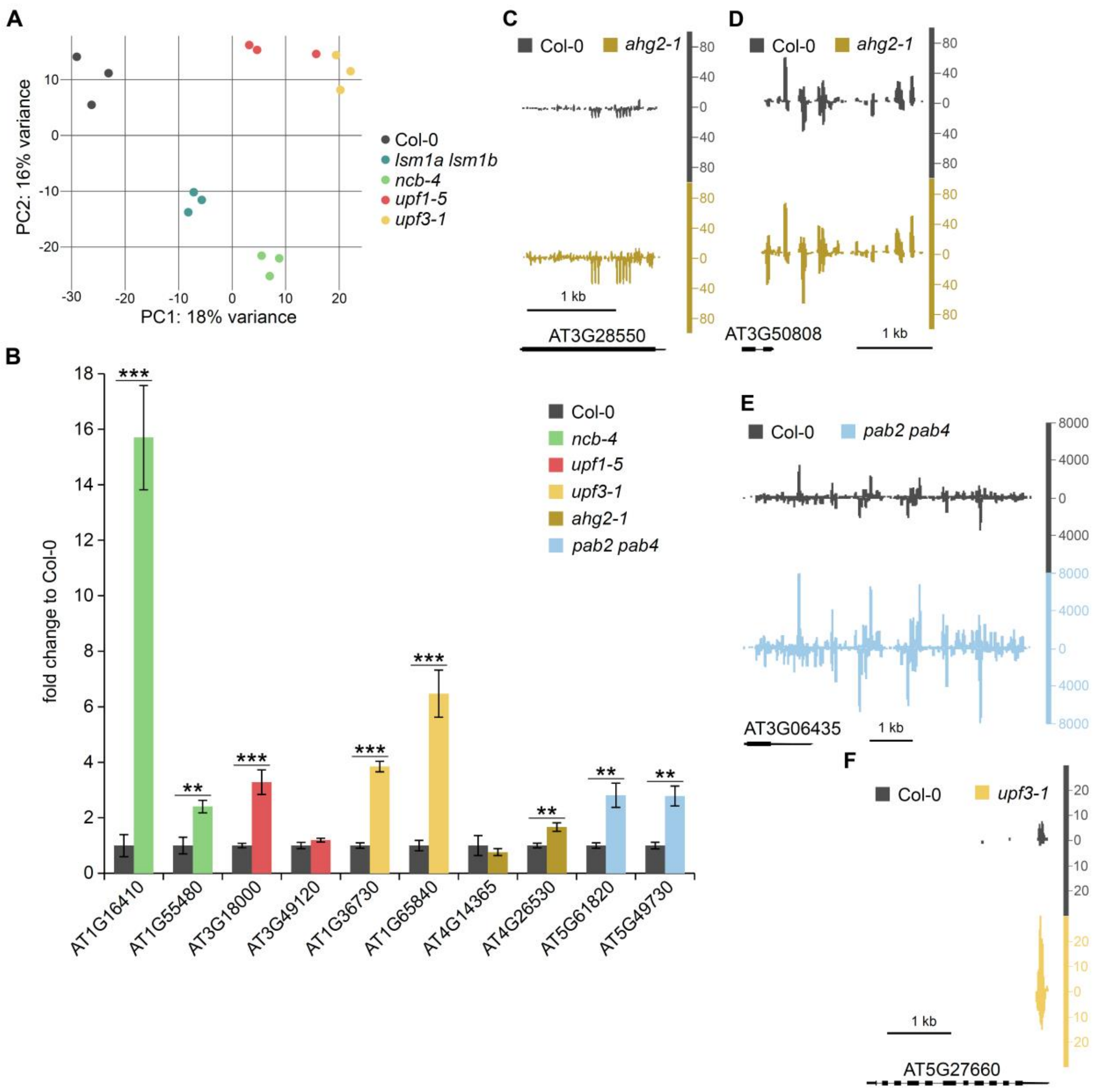


**Figure S3**. **(A)** PCA plot small RNA-seq for the second set of mutants. **(B)** RT-qPCR for selected genes with siRNAs produced only from one strand in *ncb-4*, *upf1-5*, *upf1-3*, *ahg2-1* and *pab2 pab4* mutants. Fold changes, expressed relative to the wild-type, represent a mean of three independent biological replicates with standard deviations (s.d); $^{**}P < 0.01$; $^{***}P < 0.001$ (t-test). *UBC9* mRNA was used as a reference. **(C-F)** Examples of genes with siRNA profiles lacking features of genuine rqc-siRNAs: **(C)** and **(F)** siRNAs with high level also in the wild type, **(D-E)**: intergenic siRNA hotspots that overlap the gene only partially. Small RNAs tracks were normalised to reads per ten million.

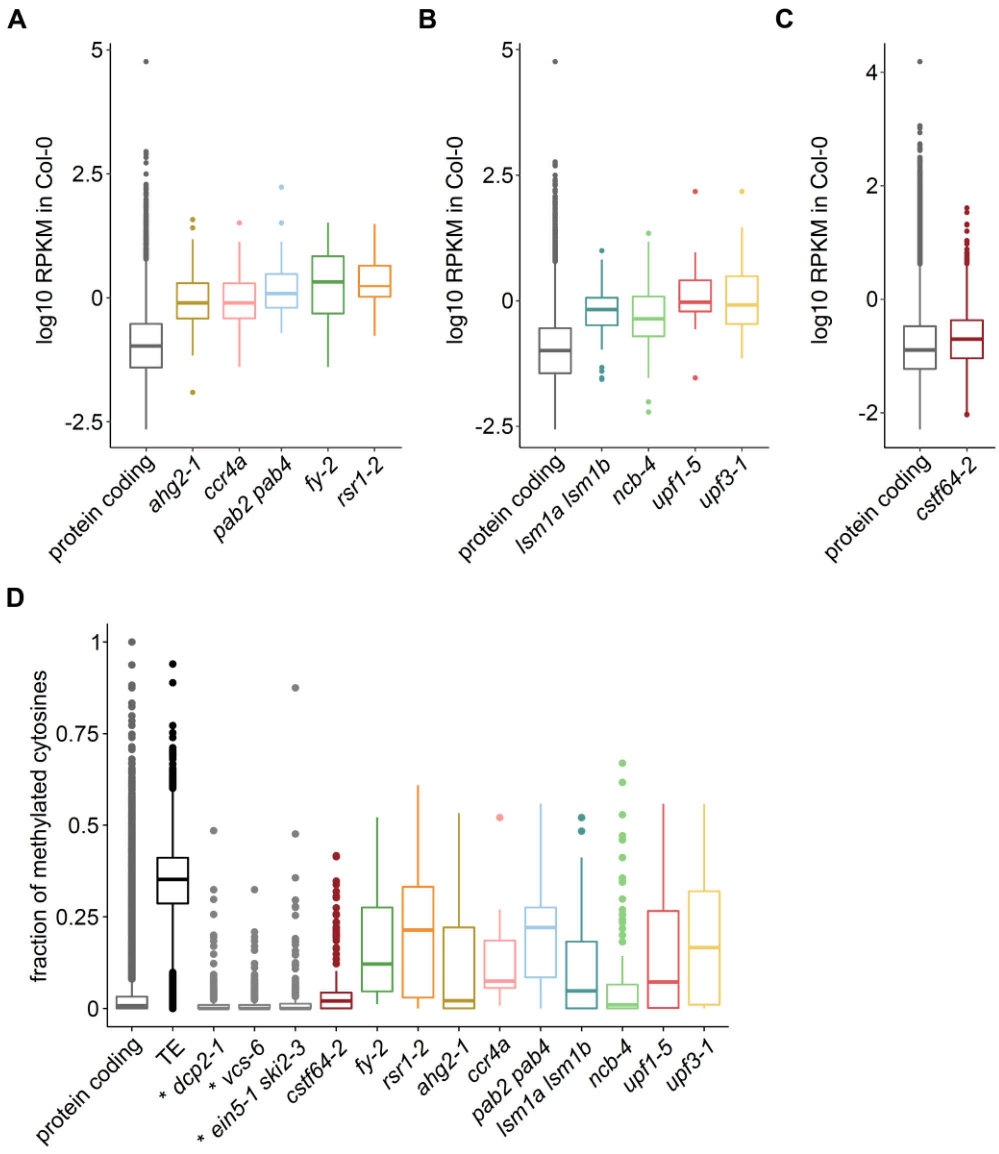


**Figure S4**. **(A-C)** In most of the analysed mutants, many genes with elevated siRNAs show a high level of small RNA production also in the Col-0 control. Small RNA levels were normalised to RPKMs. **(D)** Analysis of DNA methylation levels shows that many genes with up-regulation of siRNAs are characterised by the high level of methylated cytosines. The fraction of methylated cytosines were calculated for each gene based on published data for Col-0 (Schmitz et al. 2013). Two first boxplots show values for protein-coding genes and transposable elements (TE). The following three boxplots show results for protein-coding genes with elevated siRNA production in the published mutants: *dcp2-1* and *vcs-6* (Martinez de Alba et al. 2015), and the *ein5-1 ski2-3* double mutant (Zhang et al. 2015).

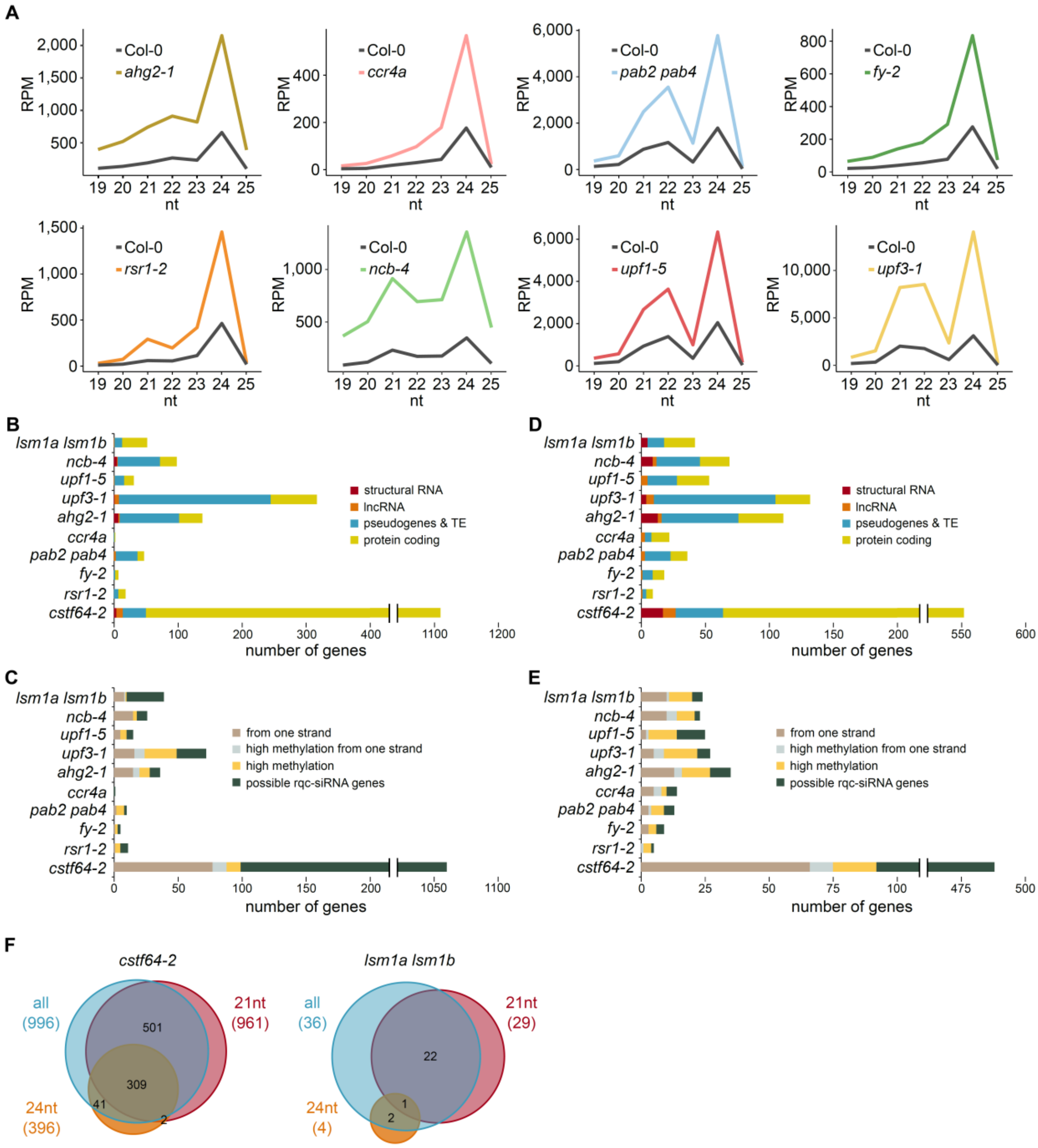


**Figure S5**. **(A)** Length distribution of siRNAs from protein-coding genes showing their up-regulation in the different analysed mutants. (**B**) Araport11-annotated genes with a significant up-regulation of 21-22 nt small RNAs ($log_2FC > 1$, $FDR < 0.05$) were divided into producing structural RNAs, long non-coding RNAs, pseudogenes and transposable elements, and protein-coding. **(C)** Protein-coding genes with the accumulation of siRNAs were divided into producing siRNAs from only one strand, genes with high DNA methylation levels (> 20% of all cytosines), genes fitting these two categories and genes that represent a possible source of rqc-siRNAs. **(D-E)** As for A-B but 24 nt long siRNAs were analysed. **(F)** Venn diagrams show a high overlap between genes identified for siRNA length classes and all siRNAs.

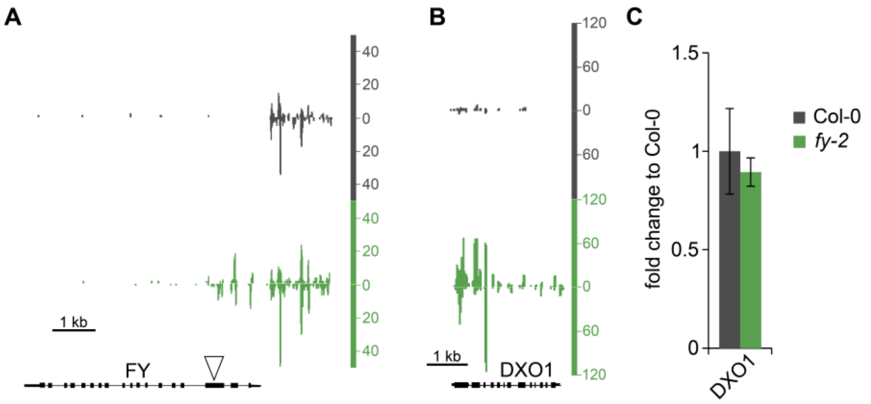


**Figure S6**. **(A)** siRNAs produced in the *fy-2* mutant downstream of the T-DNA insert (triangle) in the *FY* gene. **(B)** Profile of small RNA reads in Col-0 and the *fy-2* mutant over the *DXO1* gene. Small RNAs tracks were normalised to reads per ten million. **(C)** RT-qPCR of the *DXO1* mRNA. Fold change, expressed relative to the wild-type, represent a mean of three independent biological replicates with standard deviations (s.d.). *UBC9* mRNA was used as a reference.

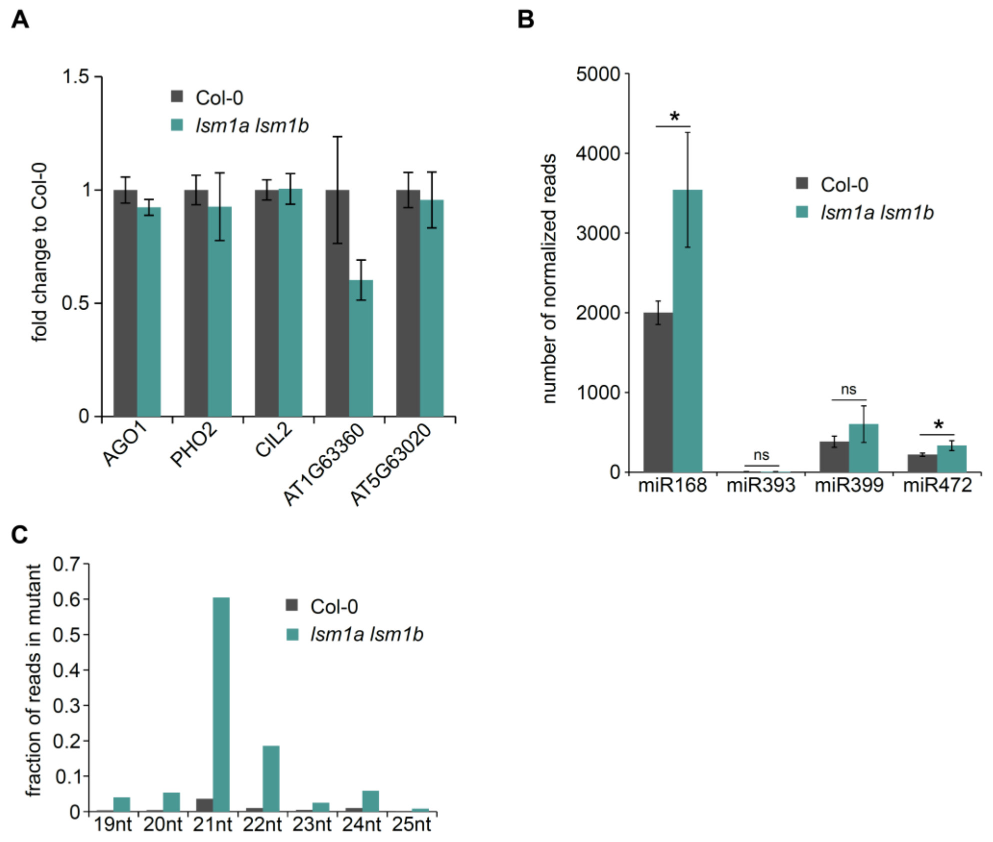


**Figure S7**. **(A)** RT-qPCR of five genes showed in profiles on Figure 4E-K. Fold changes, expressed relative to the wild-type, represent a mean of three independent biological replicates with standard deviations. None of these mRNAs showed a significant difference in their level (t-test). *UBC9* mRNA was used as a reference. **(B)** Quantification of miRNAs that target mRNAs with siRNA increase in the *lsm1a lsm1b* mutant. Bars represent mean of the normalised number of reads (RPM) from three sequencing biological replicas with standard deviations (s.d.); *$P < 0.05$, ns $P > 0.05$ (t-test). **(C)** Length distribution of rqc-siRNAs for genes showing their up-regulation in *lsm1a lsm1b* double mutant.

**Table S1.** Libraries sequencing depth.

| | library 1 (M reads) | library 2 (M reads) | library 3 (M reads) |
|---|---|---|---|
| Col-0 (1) | 11.4 | 11.2 | 11.2 |
| *lsm1a lsm1b* | 10.9 | 11.3 | 11.1 |
| *ncb-4* | 11.4 | 11.1 | 10.3 |
| *upf1-5* | 11.1 | 11.1 | 11.2 |
| *upf3-1* | 11.4 | 10.8 | 11.0 |
| Col-0 (2) | 12.6 | 13.4 | 13.2 |
| *ahg2-1* | 13.0 | 12.1 | 13.4 |
| *ccr4b* | 13.5 | 13.6 | 13.3 |
| *fy-2* | 13.2 | 13.5 | 13.4 |
| *pab2 pab4* | 13.7 | 13.6 | 13.3 |
| *rsr1-2* | 13.3 | 13.6 | 13.5 |
| Col-0 (3) | 10.4 | 10.3 | 10.2 |
| *cstf64-2* | 10.3 | 10.3 | 10.1 |

**Table S2.** List of primers used in this study.

| Gene/region | Forward | Reverse |
|---|---|---|
| *AT1G16410* | CTTGCGCGTCAAGATACCACC | AGGGTTACGACCTAGTCCAGGG |
| *AT1G36730* | GATGCCGAGGATGATGACCAAG | CCAAGGCCTTAGCAATCTCAACC |
| *AT1G36990* | GCCCAAGTGGTCTTAACATGGC | CAAATCCTCGAGCCGTTGTGTC |
| *AT1G55480* | AACCGGAAAGTTCACTGTTGGC | GTACTCAGCTGCAGGCCAAATC |
| *AT1G63360* | TGATGTTTCATTGGACCTCAACAC | TTGAACCAAACCTGAATCATAAACC |
| *AT1G65840* | GATATCGGGTCTCGGTGTAGGC | CCACATGCGTAAGAAGTCGGTG |
| *AT1G68520* | CTTTGTAGAGAGAACCCATAGTTCG | CTCCAACCGCACTAGCCAAAC |
| *AT1G76170* | AGAGATTCACCAAGTCATTGTTGGG | CCGGAGGCACCTATAGCAACTC |
| *AT1G76180* | GTGGTTGTTCCTCCTCCTGTGG | CGGTGGTCTTAGGGTGGTATCC |
| *AT1G78310* | GGTTCCCTGTATCTCCGACGAC | CTTTGACCGACAAGGGTTTCCG |
| *AT2G22640* | GTGCAAGTAAGTACCGCGACAG | CCAAAGGATCAGGCTCGGTTTC |
| *AT2G36800* | CATTGAGTCTGGCTTGCCCATC | TCATCCGCTCCATTGTGTCAAG |
| *AT2G38820* | GGTACCTTACCCGGAGACGATG | GAACTGACGCTCAAAGAGACGC |
| *AT2G41430* | TGAGAATGGCGATGGTATCAGG | AGGGTACCAAGTCGAAGTTGTC |
| *AT2G41460* | TCGTTGGAGTTGGAGTGGGAAC | TGAGTTCCTTTCGCTTGCTGTTG |
| *AT2G45180* | TGAAACCCACTTGTCCTACCG | GGTGGAGAACCAACAACAACG |
| *AT2G46820* | TGTTTCTGTGAGATTCTTCCGAGGC | AGAAGAGGCGGAGGCGGAGG |
| *AT3G11440* | CTTCTTGGTGGTGACGACATTGG | TTGGTCCATCCATAAGACCCGAC |
| *AT3G18000* | AGGATGGTCGGTTGGATCAAGG | TTGGTGGAAGCAAGATTCACGG |
| *AT3G32940* | TGAGGCCGTCGACCCTATTTAC | GAGCCAAAGGCCGTACTGATTG |
| *AT3G49120* | AAATGCCTCCAGCTTTGACCC | ACCCGAGAAGATGAGGAAAGTC |
| *AT3G59540* | AAGGAAGGATGCAAGGTCTGTG | CTTATCGGCCTTCTCTTGGTCG |
| *AT3G61630* | CTCCGAGACTGGTCCGTATCAC | CGTTTCACTCTTGGAACCACCG |
| *AT3G63390* | CAAAGACAAGTGCTCTGGTGCC | CTGCTTTCCTCCGGCATTGAAG |
| *AT4G08455* | CCGTTCTGGTGAGTCGTTCTCC | CGTGCCGCTTAGACTCTCTTCC |
| *AT4G14365* | AGTAATGTCGTACGCGCAATCG | AGCTGGAGCCAGAATACTCACG |
| *AT4G26530* | TCACCCATTTGCTTTATCCGAGG | TGGAGTGCAAGAGAGACGAG |
| *AT5G49100* | ACACCAAGTGAGAGGAAAGTGTCG | AGCTTCTACTTCCACACCCAACAG |
| *AT5G49730* | AACGATGCTTTCAAGGCCAAAG | ACACATATCAACCATAGCTGTTTCC |
| *AT5G61820* | AACCAGCTTCGTTTCCAGGTTC | CGGTGGTGCTCTATACCGAGAG |
| *AT5G63020* | AGGATGGGAGGTTGTGTCTCTG | AGCTGTCAGATTCTCCTCTAAGCC |
| *RT_AT1G37130* | TCGTGAATCTGTTGTTGGTAACG | GCATCACAGTAGATCGACCAATAAG |
| *RT_AT1G68520* | TCCCAAGCCAAGTACTCTTCTAC | GTTGAGGGACACTTGAACAAAC |
| *RT_AT1G76170* | ACTATACGATGTCGTTTCTGGTTC | TGGATTAGGTGTTCCATGTGTGG |
| *RT_AT1G76180* | TGTAGACCAGTTGACGTAACGTTTCCC | ACCCACAATGATGAGCGTTAAAGACAT |
| *RT_AT2G38820* | TGTGACATTTGGTAAATCAGAGAGA | GCCAGAAACGACCACATCAAGA |
| *RT_AT2G41430* | AGAACTCTACAAGGTTGGTGGC | GAAATGAACTTGGTGTCGTCGG |
| *RT_AT2G41460* | CACAAGCTTTATACTCGTGCGG | GTCATACCGGCTGATCCAATTC |
| *RT_AT2G45180* | TGAGAAGCAATTTCCGTTGGG | CCTTAGCAATCAGTGGGCCTG |
| *RT_AT2G46820* | AATAGCGCGTGTAGTGAACCGC | CACTAGAAACATCAGTGGACCCGG |
| *RT_AT3G63390* | GGTGCAAACACTCTTGTTTCTTTC | TGAAGTGAATGTCGAGAAACTCC |
| *ACT2 (AT3G18780)* | GGTAACATTGTGCTCAGTGG | CTCGGCCTTGGAGATCCACA |
| *AGO1 (AT1G48410)* | GGAAGCTCTGCAGGTTCTTGAC | ATACCTGATTCTAGAGGTCGGC |
| *CIL2 (AT3G23690)* | CACAACCAACAACGCAGGAGAG | TTCGTGGTGTGAACTCGATCCG |
| *DXO1 (AT4G17620)* | CGGCGAAGACTCCGACAACG | TCCGCACCACCTCCATCTCC |
| *NIA1 (AT1G77760)* | GCACCTCTTCCTCGTCTTATGC | AATTCGCTTTGGGAACTGCACC |
| *NIA2 (AT1G37130)* | ACGATTCCTTATGGATCACCCG | TCAAACTCCTCCGTACAATCCG |
| *PHO2 (AT2G33770)* | AAAGGATGGACCTTCTGCGTGC | AGCCCATCGTGATATGGCGTTC |
| *SMXL5 (AT5G57130)* | TGGGCACTTCATCCTGTCTCTG | CTTGCCTCATGTCCACTTGTCG |
| *UBC9 (AT4G27960)* | TTCATGTAGCGCAGGACCCGTTG | ACTCCTCCAGAATAAGGGCTATCCG |
